# Non-targeted metabolomics and lipidomics LC–MS data from maternal plasma of 180 healthy pregnant women

**DOI:** 10.1186/s13742-015-0054-9

**Published:** 2015-04-09

**Authors:** Hemi Luan, Nan Meng, Ping Liu, Jin Fu, Xiaomin Chen, Weiqiao Rao, Hui Jiang, Xun Xu, Zongwei Cai, Jun Wang

**Affiliations:** 1 Department of Chemistry, Hong Kong Baptist University, 224 Waterloo Road, Kowloon Tong, Hong Kong China; 2 BGI-Shenzhen, Building No.11, Beishan Industrial Zone, Yantian District, Shenzhen 518083 China; 3 Department of Biology, University of Copenhagen, Ole Maaløes Vej 5, DK-2200 Copenhagen, Denmark

**Keywords:** Metabolomics, Lipidomics, Pregnancy, Metabolic phenotype, Maternal plasma

## Abstract

**Background:**

Metabolomics has the potential to be a powerful and sensitive approach for investigating the low molecular weight metabolite profiles present in maternal fluids and their role in pregnancy.

**Findings:**

In this Data Note, LC–MS metabolome, lipidome and carnitine profiling data were collected from 180 healthy pregnant women, representing six time points spanning all three trimesters, and providing sufficient coverage to model the progression of normal pregnancy.

**Conclusions:**

As a relatively large scale, real-world dataset with robust numbers of quality control samples, the data are expected to prove useful for algorithm optimization and development, with the potential to augment studies into abnormal pregnancy. All data and ISA-TAB format enriched metadata are available for download in the MetaboLights and *GigaScience* databases.

**Electronic supplementary material:**

The online version of this article (doi:10.1186/s13742-015-0054-9) contains supplementary material, which is available to authorized users.

## Data description

### Purpose of collection

**Figure 1:**
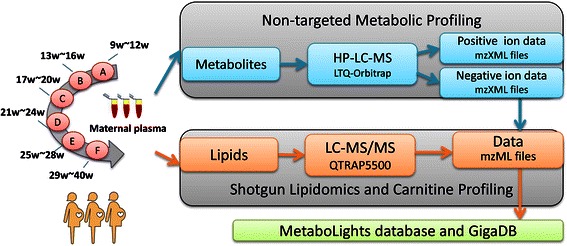
**An overview of the three datasets for the different experiments.**

Metabolic variations occur during normal pregnancy to provide the developing foetus with a supply of nutrients required for development, and to ensure the health of the mother during gestation. The following dataset was collected to study metabolic phenotype variations in the maternal plasma, which are induced by pregnancy during each of its three trimesters [1]. All work described here is associated with this report, which used liquid chromatography–mass spectrometry (LC–MS) to survey the effects of pregnancy on the metabolite profiles of maternal plasma during pregnancy. A basic overview of the three datasets for the different experiments is shown in Figure 1. Full details of the batch numbers, run order and distribution of QCs can be seen in Additional file 1.

### Sample collection

A total of 180 healthy pregnant women were recruited from the Maternity and Child Health Hospital of Shenzhen, China. Full ethical approval for this study was provided by the Institutional Review Board of BGI-Shenzhen (No. 13045). All volunteers gave written informed consent and filled out an individual questionnaire at the time of collection for the study. Clinical information was obtained from neonatal and obstetrical medical records. Pregnancy was followed up to term to ensure participating women had normal term pregnancies and healthy babies. Individual participants were divided into six subgroups of 30 individuals according to their gestational weeks: class A (9w ~ 12w), class B (13w ~ 16w), class C (17w ~ 20w), class D (21w ~ 24w), class E (25w ~ 28w), and class F (29w ~ 40w). Weight, height, age and gestational week were recorded. Venous blood was collected in the morning before breakfast using VACUETTE® EDTA Blood Collection Tubes (Greiner Bio-One GmbH, NC, USA). Plasma samples were separated at 2200 *g* for 5 min at 4°C from vein blood and stored at −80°C until use. One sample was collected per patient, rather than multiple collections for each subject.

### Non-targeted metabolic profiling spectral acquisition

Low molecular weight metabolites (<1000 Da) in the plasma samples were isolated using methanol precipitation with slight modifications to previously reported protocols [1,2]. To outline these briefly, 50 μl of thawed plasma was collected and precipitated using 200 μl of methanol. After centrifugation at 14000 *g* for 10 min at 4°C, the supernatant was transferred to 1.5 mL polypropylene tubes, and 10 μL of the supernatant injected into the LC–MS equipment. Quality control (QC) samples were also prepared by mixing equal volumes (10 μL) from each plasma sample before sample preparation as they were being aliquoted for analysis. This pooled sample was then used to estimate a mean profile representing all the analytes encountered during the analysis [3-4].

LC–MS data was acquired using a Shimadzu Prominence HPLC system (Shimadzu, Japan), coupled online to a LTQ Orbitrap Velos instrument (Thermo Fisher Scientific, MA, USA) set at a resolution of 30,000 (at *m/z* 400). Sample analysis was carried out in both positive and negative ion modes with the mass scanning range at 50–1500 *m/z* and capillary temperature of 350°C. Nitrogen sheath gas was set at a flow rate of 30 L/min, and auxiliary gas was set at a flow rate of 10 L/min. Spray voltage was 4.5 kV, with the LC–MS system run in binary gradient mode. Solvent A was 0.1% (v/v) formic acid/water and solvent B was 0.1% (v/v) formic acid/methanol; the flow rate was 0.2 ml/min. A C-18 column (150 × 2.1 mm, 3.5 μm, Agilent, USA) was used for all analysis. The following gradient was used: 5% B at 0 min, 5% B at 5 min, 100% B at 8 min, 100% B at 9 min, 5% B at 18 min and 5% B at 20 min.

The positive ion dataset was run as two batches, with the pooled QC sample repeated once every sixth sample. This produced final spectra as follows: 30 class A; 28 class B; 30 class C; 30 class D; 30 class E; 30 class F; and 39 QC samples spread throughout. The negative ion dataset was run as five batches, with the pooled QC sample injected five times at the start of the run, three times at the end, and once every five samples. The negative ion data has final spectral numbers of: 28 class A; 26 class B; 27 class C; 29 class D; 26 class E; 25 class F; and 66 QC samples spread throughout. Additionally, three blank samples were injected before the starting QC of each run for baseline stabilisation of the LC–MS system – these blank spectra are not included in these published data. Discrepancies with sample numbers are due to some samples being used up entirely as repeats were performed to correct for failed spectra. The positive and negative ion mode LC–MS data were collected in two batches and five batches, respectively, due to an observation that the negative ion responses decreased faster than positive ion responses.

### Shotgun lipidomics and carnitine profiling spectral acquisition

Lipidomics analysis was carried out as previously described, with slight modifications to previously reported protocols [5,6]. Plasma samples were thawed and extracted, with 10 μL of plasma placed in 1 mL glass vials. Eight μL of an internal standards mixture consisting of ceramide (Cer, d18:1/17:0), phosphoethanolamine (PE, 17:0), phosphatidylserine (PS, 17:0), l-α-phosphatidylinositol (Soy PI), phosphatidylcholine (PC, 18:3), and phosphoglycerol (PG, 17:0), with a concentration of 10 μM for each lipid, was added. Individual lipids were purchased from Avanti Polar Lipids (Alabaster, AL). Following this, 800 μL of chloroform/methanol/water (20:10:1, V/V/V) containing 300 mM ammonium acetate was added and vortexed. After 10 min, centrifugation at 14000 *g* and 10°C (Eppendorf, Hamburg, Germany) was performed and 100 μL of the lower organic layer was diluted with 100 μL of methanol/chloroform (2:1, V/V) containing 10 mM ammonium acetate. The final mixture was transferred into a new vial and frozen at −20°C until required.

Lipidome and carnitine profiling were detected by multiplexed precursor ion scanning (MPIS) analysis. One hundred μL of final extract was loaded into 96 well plates (Eppendorf, Hamburg, Germany) and sealed with aluminium foil. The final extracts were analysed using a QTRAP® 5500 System equipped with a Shimadzu Prominence HPLC system (Shimadzu, Japan). Liquid chromatography and flow injection analysis–mass spectrometry were employed to acquire the data. The electrospray source was operated in the positive ion mode, with optimised source parameters: ionSpray Voltage 5.5 kV, source temperature 300°C, curtain gas 30. Collision energies ranged from 25–30 V for various lipid species in precursor ion or neutral loss scan modes. Flow injection analysis was performed with a mixture of chloroform-methanol-10 mM ammonium acetate in water (300/665/35, V/V/V) at a flow rate of 200 μl/min. Total analysis time per sample was 3.6 min. PE, PC, PS, PI, PG, Cer and carnitines were monitored by consecutive positive ion mode PIS *m/z* 141.0, 184.1, 185.0, 277.0, 189.0, 264.2 and 85.1, respectively. Acquired spectra were processed by LipidView™ and ChemoView™ software (AB Sciex) for isotope correction, identification and quantification of detected lipid and carnitine species as previously described [5-7].

This lipidomics dataset was run as two batches, with the pooled QC sample injected three times at the start, three times at the end and once every 20 samples. This dataset comprises: 29 class A; 29 class B; 30 class C; 30 class D; 30 class E; 29 class F; 14 QC samples throughout.

### Potential uses

On top of the already published work characterising metabolic phenotype variations during pregnancy, these data also provide a useful baseline and reference dataset to be compared to specific conditions (e.g. hypertensive disorders, diabetes mellitus) in pregnancy. The data were observed to be of good quality via plotting (PCA scores) of QCs alongside samples and noting that the QC samples were tightly clustered. This was observed after processing, full details of which can be found in the previously published research article [1]. Although the positive ion, non-targeted dataset was not randomised during LC–MS collection, the inclusion of QC samples throughout the collection process arguably allows for correction of drift and other systematic noise (e.g. biases correlated with analysis order and/or sample preparation order). The other two datasets were fully pseudo-randomised. The large scale of this dataset and the inclusion of quality control measures makes it very useful for algorithm optimisation and development, especially those that deal with quality control issues such as batch and drift correction [8-9].

The lipidomics and carnitine dataset ought to be of particular use as a biological reference when studying abnormal pregnancy due to its ability to describe nutrition and energy levels in the subject.

## Availability of supporting data and materials

Supporting data and corresponding ISA-TAB metadata are available in the MetaboLights database [MTBLS146], as well as the *GigaScience* GigaDB repository [10].

## Additional file

Additional file 1:
**A detailed, graphical depiction of batch numbers, run order and distribution of QCs for all datasets.**

## Abbreviations

BMI: Body mass index
LC: Liquid chromatography
MS: Mass spectrometry
PC: Phosphatidylcholine
PE: Phosphoethanolamine
PS: Phosphatidylserine
PI: Phosphatidylinositol
PG: Phosphoglycerol
Cer: Ceramide
