# Supplementary figures and images for: Non-targeted metabolomics and lipidomics LC–MS data from maternal plasma of 180 healthy pregnant women
Source: Gigascience. 2015 Apr 9;4:16. doi: 10.1186/s13742-015-0054-9 (PMC4391677; doi:10.1186/s13742-015-0054-9)

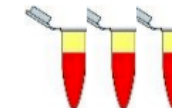[illegible][illegible][illegible][illegible]

Batch 1Batch 2

Supplement: Additional file 1: — A detailed, graphical depiction of batch numbers, run order and distribution of QCs for all datasets. [file 13742_2015_54_MOESM1_ESM.pdf]
